# CottonMD: a multi-omics database for cotton biological study

**DOI:** 10.1093/nar/gkac863

**Published:** 2022-10-10

**Authors:** Zhiquan Yang, Jing Wang, Yiming Huang, Shengbo Wang, Lulu Wei, Dongxu Liu, Yonglin Weng, Jinhai Xiang, Qiang Zhu, Zhaoen Yang, Xinhui Nie, Yu Yu, Zuoren Yang, Qing-Yong Yang

**Affiliations:** National Key Laboratory of Crop Genetic Improvement, College of Informatics, Huazhong Agricultural University, Wuhan 430070, China; Hubei Key Laboratory of Agricultural Bioinformatics, College of Informatics, Huazhong Agricultural University, Wuhan 430070, China; Innovative Center of Molecular Genetics and Evolution, School of Life Sciences, Guangzhou University, Guangzhou 510405, China; National Key Laboratory of Crop Genetic Improvement, College of Informatics, Huazhong Agricultural University, Wuhan 430070, China; Hubei Key Laboratory of Agricultural Bioinformatics, College of Informatics, Huazhong Agricultural University, Wuhan 430070, China; National Key Laboratory of Crop Genetic Improvement, College of Informatics, Huazhong Agricultural University, Wuhan 430070, China; Hubei Key Laboratory of Agricultural Bioinformatics, College of Informatics, Huazhong Agricultural University, Wuhan 430070, China; National Key Laboratory of Crop Genetic Improvement, College of Informatics, Huazhong Agricultural University, Wuhan 430070, China; Hubei Key Laboratory of Agricultural Bioinformatics, College of Informatics, Huazhong Agricultural University, Wuhan 430070, China; National Key Laboratory of Crop Genetic Improvement, College of Informatics, Huazhong Agricultural University, Wuhan 430070, China; Hubei Key Laboratory of Agricultural Bioinformatics, College of Informatics, Huazhong Agricultural University, Wuhan 430070, China; National Key Laboratory of Crop Genetic Improvement, College of Informatics, Huazhong Agricultural University, Wuhan 430070, China; Hubei Key Laboratory of Agricultural Bioinformatics, College of Informatics, Huazhong Agricultural University, Wuhan 430070, China; Hubei Key Laboratory of Agricultural Bioinformatics, College of Informatics, Huazhong Agricultural University, Wuhan 430070, China; Hubei Key Laboratory of Agricultural Bioinformatics, College of Informatics, Huazhong Agricultural University, Wuhan 430070, China; National Key Laboratory of Crop Genetic Improvement, College of Informatics, Huazhong Agricultural University, Wuhan 430070, China; Hubei Key Laboratory of Agricultural Bioinformatics, College of Informatics, Huazhong Agricultural University, Wuhan 430070, China; State Key Laboratory of Cotton Biology, Institute of Cotton Research, Chinese Academy of Agricultural Sciences, Anyang 455000, China; Key Laboratory of Oasis Ecology Agricultural of Xinjiang Bingtuan, Agricultural College, Shihezi University, Shihezi, Xinjiang 832000, China; Xinjiang Academy of Agricultural and Reclamation Science, Shihezi, Xinjiang 832000, China; State Key Laboratory of Cotton Biology, Institute of Cotton Research, Chinese Academy of Agricultural Sciences, Anyang 455000, China; Xinjiang Academy of Agricultural and Reclamation Science, Shihezi, Xinjiang 832000, China; National Key Laboratory of Crop Genetic Improvement, College of Informatics, Huazhong Agricultural University, Wuhan 430070, China; Hubei Key Laboratory of Agricultural Bioinformatics, College of Informatics, Huazhong Agricultural University, Wuhan 430070, China; Key Laboratory of Oasis Ecology Agricultural of Xinjiang Bingtuan, Agricultural College, Shihezi University, Shihezi, Xinjiang 832000, China; Xinjiang Academy of Agricultural and Reclamation Science, Shihezi, Xinjiang 832000, China

## Abstract

Cotton is an important economic crop, and many loci for important traits have been identified, but it remains challenging and time-consuming to identify candidate or causal genes/variants and clarify their roles in phenotype formation and regulation. Here, we first collected and integrated the multi-omics datasets including 25 genomes, transcriptomes in 76 tissue samples, epigenome data of five species and metabolome data of 768 metabolites from four tissues, and genetic variation, trait and transcriptome datasets from 4180 cotton accessions. Then, a cotton multi-omics database (CottonMD, http://yanglab.hzau.edu.cn/CottonMD/) was constructed. In CottonMD, multiple statistical methods were applied to identify the associations between variations and phenotypes, and many easy-to-use analysis tools were provided to help researchers quickly acquire the related omics information and perform multi-omics data analysis. Two case studies demonstrated the power of CottonMD for identifying and analyzing the candidate genes, as well as the great potential of integrating multi-omics data for cotton genetic breeding and functional genomics research.

## INTRODUCTION

Cotton is an important economic crop in the world. More than 50 species have been found in the cotton genus (*Gossypium*), among which upland cotton (*G. hirsutum*, AADD, 2*n* = 4*x* = 52) accounts for over 90% of the world's cotton lint production (1). With the great advances in genome sequencing technology and computing power, many studies of genome assembly and population genetics in cotton have been carried out, resulting in the identification of numerous variations associated with important traits using the statistical methods, such as genome-wide associated study (GWAS) (2–11). However, the majority of these associations cannot be reasonably explained. Currently, rapid advances in transcriptomics, epigenomics and metabolomics of cotton have led to the accumulation of large amounts of high-dimensional ‘omics’ biological data, making it possible to uncover the functions of these variants (12,13). Several genomic, transcriptomic, genetic variation, epigenetic databases have been constructed and released in the cotton genus, such as CottonGen (14), CottonFGD (15), ccNET (16), and MaGenDB (17), CottonGVD (18) and GRAND (19). CottonGen provides genomics, genetic and breeding data including genome sequences, genes, unigenes, markers, trait loci, genetic maps and germplasm resources (14). CottonFGD integrates genome sequences and annotations, genetic markers, and gene expression and sequence variation data for four sequenced *Gossypium* species (15). ccNET contains multi-dimensional co-expression networks across mutiple *Gossypium* species (16). MaGenDB contains functional annotations and genome browser of diverse omics datasets for 13 *Malvaceae* species (17). CottonGVD contains the genomic information, population variations, and the visualized tools of GWAS results from three cultivated cotton species (18). GRAND integrates 18 cotton genome sequences, genome annotations, two cotton genome variations information and four transcriptomes for Gossypium species (19). These databases provide abundant cotton multi-omics data sources, but there is a lack of comprehensive integration and platform of multi-layer omics datasets to facilitate more systematic analyses, sophisticated understanding of the interesting genes and genetic breeding without switching to different databases.

In this study, we constructed a cotton multi-omics database (designated as CottonMD, http://yanglab.hzau.edu.cn/CottonMD/) by mining and integrating the data of 25 genomes, transcriptomes (from 76 different tissue samples), genetic variations (from 4180 accessions), phenotypic data (from 20 phenotypes), epigenomes (from five species) and 768 metabolites (from four tissues). CottonMD provides a large amount of multi-omics data and easy-to-use tools, which will be a valuable database for future cotton genetic breeding and functional genomics research.

## MATERIALS AND METHODS

### Data sources

**Figure 1.**
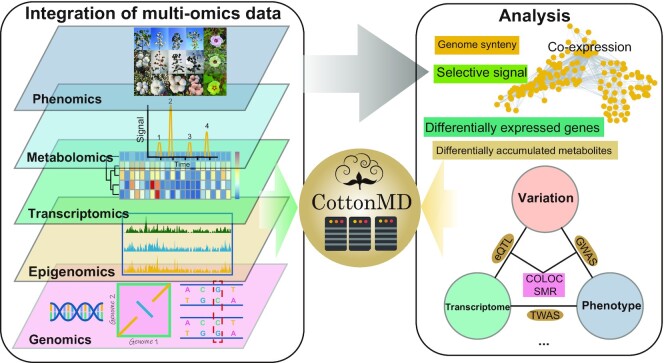
Overview of CottonMD. Construction pipeline of CottonMD through integration of multi-omics data.

**Figure 2.**
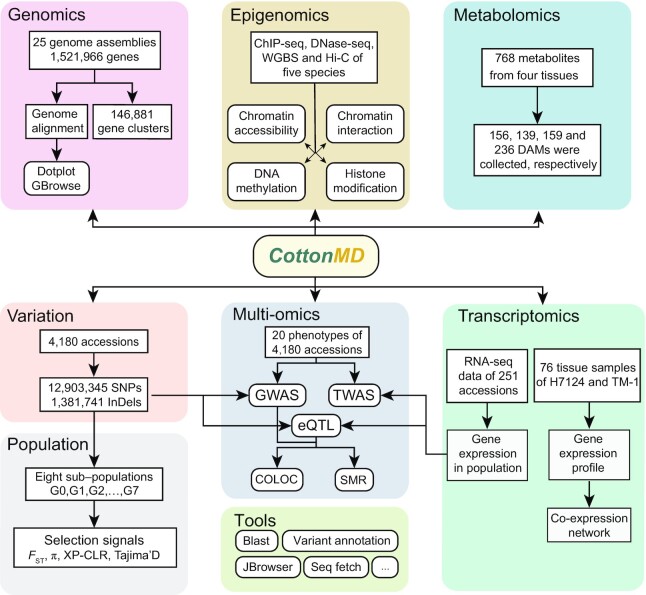
Basic schema and data source of CottonMD. The rounded rectangles with different colors indicate different portals.

To construct a comprehensive cotton multi-layer omics platform, we mined and integrated the data from genomics, transcriptomics, genetic variation, phenotypic data, epigenetics and metabolomics (Figure 1; Supplementary Tables S1–S4). In total, 25 genome assemblies and 1 826 891 genes of 16 germplasms including the diploids A_1_-, A_2_-, D_1_-, D_5_-, D_10_-genomes and allopolyploids (AD)_1_-, (AD)_2_-, (AD)_3_-, (AD)_4_- and (AD)_5_-genomes were collected from public databases (Supplementary Table S1). As for phenotypic data, 20 phenotypes were collected from six studies (2,4,5,20–22) (Figure 2; Supplementary Table S2). Transcriptome data from different tissues and individuals was listed in Supplementary Table S3. Epigenetics data are summarized in Supplementary Table S4. Metabolome datasets were retrieved from two previous studies (23,24). Genome resequencing data from a total of 4180 accessions including 3743 *G. hirsutum*, 393 *G. barbadense*, seven *G. tomentosum*, six *G. darwinii*, six *G. mustelinum* and 25 other accessions were mainly collected from previously published studies (2,4,5,20,25–31) (NCBI BioProject accession number: PRJNA257154, PRJNA336461, PRJNA375965, PRJNA399050, PRJNA414461, PRJNA473334, PRJNA530048, PRJNA576032 and PRJNA605345) (Supplementary Table S5).

### Comparative genome analysis

Genome sequences were compared between TM-1 reference genome and other 25 genome assemblies using the NUCmer program (v.4.0.0beta2) with parameters ‘nucmer – maxmatch –noextend’ in MUMmer4 (32). After filtering of the one-to-one alignments with a minimum alignment length of 50 bp using the show-diff program from MUMmer4, the remaining alignment blocks were used for genome browser visualization (32). For genome browser visualization, dotplot module in JBrowse2 and Genome synteny module in Gbrowser were embedded in CottonMD (33,34).

A total of 1 826 981 genes from 25 genome assemblies were used to construct the gene clusters. Firstly, protein sequences of every pair from 25 genome assemblies were aligned using diamond (v.0.9.14.115, http://github.com/bbuchfink/diamond). Then, gene synteny was detected by McScan (python version) (35). The genes with gene synteny were grouped to one cluster. Finally, 1 521 966 genes were grouped to 146,881 gene clusters.

### SNP and InDel calling

The genome resequencing data of each accession were mapped to the TM-1 reference genome using BWA-MEM with default parameters (11,36). Then, the reads with the mapping quality value <20 were removed by SAMtools (v.1.6) (37). SNPs and small InDels were identified using Sentieon DNAseq pipeline for each accession (38). SNPs with low mapping quality were filtered out by GATK VariantFiltration with parameters ‘QUAL < 30.0 || MQ < 50.0 || QD < 2’ (39). All SNPs and InDels with minor allele frequencies (MAF) <0.01 or missing rate >0.1 were discarded by VCFtools (v.0.1.16) (40). As for the remaining SNPs and InDels, genotype imputation was performed using beagle (v.5.1) (41).

### Transcriptome analysis

After clipping the adaptor sequences and removing the low-quality reads by Trimmomatic software (v.0.36) (42), the RNA-seq clean data from accessions were mapped to the TM-1 reference genome using Hisat2 (v.2.1.2) with default parameters (43). Gene expression level was normalized using the number of transcripts per kilobase million reads (TPM) by StringTie software (v.1.3.5) with default settings (44). The co-expression network was obtained by calculating the Pearson correlation coefficient of pairwise gene expression levels, and the gene modules including the gene pairs with a Pearson correlation coefficient of larger than 0.8 were retained as a co-expression network.

### Epigenome analysis

The adaptor sequences were removed and the low-quality reads were filtered out using Trimmomatic (v.0.36). As for ChIP-seq and ATAC-seq, the clean data from accessions were mapped to the TM-1 reference genome using bowtie2 (v.2.3.2) with default parameters (45). PCR duplicated reads were removed using Picard tools (v.2.19). Peaks were called using the callpeak module of MACS2 software (v.2.1.2) with the parameters ‘ –broad -f BAM -g 2290000000 -B -p 0.00001 –nomodel –extsize 147 ’ (46).

As for Hi-C, the clean reads of each accession were mapped to the TM-1 reference genome using BWA-MEM with default parameters (11,36). Then, the Hi-C interaction matrix was created using Juicer pipeline (47). KR normalized matrix was extracted from Hi-C format files at the resolutions of 10 kb, 50 kb and 100 kb using Juicer_tools (v.1.7.6) for JBrowser (34,48).

As for BS-seq data, clean data of each accession were mapped to the TM-1 reference genome using Bismark (v.0.13.0) with parameter settings ‘-N 1, -L 30’ (49). Bigwig files of all epigenome data analysis can be visited by JBrowser in the Tools portal (34).

### Population genetic analysis

SNPs and InDels were filtered based on linkage disequilibrium (LD) using PLINK (v.1.90b4.4) with the parameters ‘–indep-pairwise 100 50 0.8’ (50). Variations passing filtering were used for the downstream analysis. Phylogenetic tree of 4180 cotton accessions was constructed using FastTreeMP (v.2.1) with the default parameters (51). Population structure of all accessions was analyzed using fastStructure with K from 2 to 10 (52). Principal component analysis (PCA) was performed using GCTA (v.1.92.4 beta2) (53).

For each subpopulation, we calculated the level of genetic diversity (π) and Tajima's *D* statistic in each 100-kb interval across the cotton genome by VCFtools (40). We calculated the level of population differentiation between cultivated populations and landraces, wild varieties and island cotton populations using *F*_ST_ with 100-kb windows sliding 20 kb by VCFtools (40). We also used the XP–CLR method to scan for domestication-sweep regions (–maxsnps 600 –size 50000 –step 10000) (54).

### Genome-wide association study (GWAS)

The SNPs and InDels with a minor allele frequency (MAF) of lower than 5% were filtered for genome-wide association study (GWAS). GWAS was performed for six traits using the GEMMA (v.0.98.1) (55). The population structure was controlled by including the first two principal components as covariates, as well as an IBS kinship matrix derived from all variants (SNPs and InDels) calculated by GEMMA. The cutoff for determining significant associations was set as –log_10_(1/*n*), where *n* represents the total number of variations.

### Expression quantitative trait loci (eQTL) mapping

The gene expression values were taken as the values of the phenotype for eQTL mapping. Only those genes expressed in more than 95% of the accessions were defined as expressed genes for eQTL mapping. Variations with MAF >5% were used to perform GWAS for each gene by using GEMMA to detect the associations for variations and genes (55). The cutoff for determining significant associations was set as –log_10_(1/*n*), where *n* represents the total number of variations. Then, eQTL mapping was performed as previously described (56). Based on the distance between eQTL and targeted-genes, we subdivided all eQTL into *cis*-eQTL if the variation was found within 1 Mb of the transcription start site or transcription end site of the target gene, otherwise as *trans*-eQTL. In CottonMD, the regulatory relationship of *trans*-eQTL was visualized using BioCircos.js (57).

### Transcriptome-wide association studies (TWAS)

TWAS was used to integrate GWAS and gene expression datasets to identify gene-trait associations. TWAS was conducted by the EMMAX module using the gene expression data of fiber at 20 DAP with the data of six phenotypes from *cis*-eQTL in the region of 1 Mb upstream to 1 Mb downstream of target genes to compute the gene expression weights (58,59). Models were considered as ‘transcriptome-wide significant’ if they passed the Bonferroni correction for all genes.

### Colocalization analysis

Colocalization of GWAS and eQTL results was performed to generate posterior causal probabilities for each of the variants in the GWAS and eQTL analyses. All variations within 1 Mb flanking region around the gene were tested for colocalization using the ‘COLOC’ R package with default parameters (60). The variants in *cis*-eQTLs of genes and QTLs of phenotypes were defined as colocalized when the posterior probability of a colocalized signal (PPH_4_) value was larger than 0.5 and there is at least one shared significant variation.

### SMR analysis

SMR analysis integrated the summary-level data from GWAS with eQTL data to identify genes associated with a complex trait because of pleiotropy. The *cis*-eQTL signals of expressed genes and GWAS signals of the phenotype were used to perform SMR analysis and HEIDI test by SMR software (v.1.03) (58). Then, the gene was defined to be a candidate gene of the phenotype when –log_10_(*P*-value) of SMR was <3.77 (1/*n*, *n* is the number of all expressed protein-coding genes) and *P*-value of HEIDI test was larger than 0.01_._

### Implementation

CottonMD (http://yanglab.hzau.edu.cn/CottonMD) was constructed based on the Flask (v.1.1.1) framework with AngularJS (v.1.6.1) as the JavaScript library, and runs on the Apache 2 web server (v.2.4.18) with MongoDB (v.3.4.2) as its database engine. The database is available online without registration and optimized for Chrome (recommended), Internet Explorer, Opera, Firefox, Windows Edge and macOS Safari.

## DATABASE CONTENT AND USAGE

### Overview of CottonMD

CottonMD is a multi-omics database which integrates genome, transcriptome, genetic variation, phenotype, epigenome and metabolite datasets and provides many easy-to-use analysis tools. CottonMD comprises eight portals: Genomics, Transcriptomics, Population, Variation, Epigenetics, Metabolome, Multiomics and Tools (Figure 2). These portals all provide the abundant and convenient visual tools for users to browse and compare the genome sequences, gene structures, epigenetic signals and metabolite contents and understand the mechanism of gene regulatory and evolution.

### Mining and browsing of omics data

In Genomics portal, sequences, transposons and genes from 25 genome assemblies were aligned and annotated. Users can visually browse global genome alignments by Dotplot and local alignments by GBrowse, which can help the genome-wide identification and analysis of structural variations (SVs). The gene structure and function description of homologous genes can be acquired in the Gene search and Gene cluster modules (***Case study 1***). In the Transcriptomics portal, the expression patterns of paralogs in different tissues and populations or under different treatment conditions and the co-expression network of genes can be queried (***Case study 1***). As for Epigenetics portal, chromatin interaction, chromatin accessibility, histone modification and DNA methylation of six germplasms were collected and analyzed. Users can browse the peaks of chromatin accessibility and histone modification, methylation levels of genes and chromatin interaction features in the corresponding modules of Epigenetics portal. As for Metabolome portal, metabolite content and differentially accumulated metabolites can be browsed by selecting the metabolite category in Metabolome portal.

**Figure 3.**
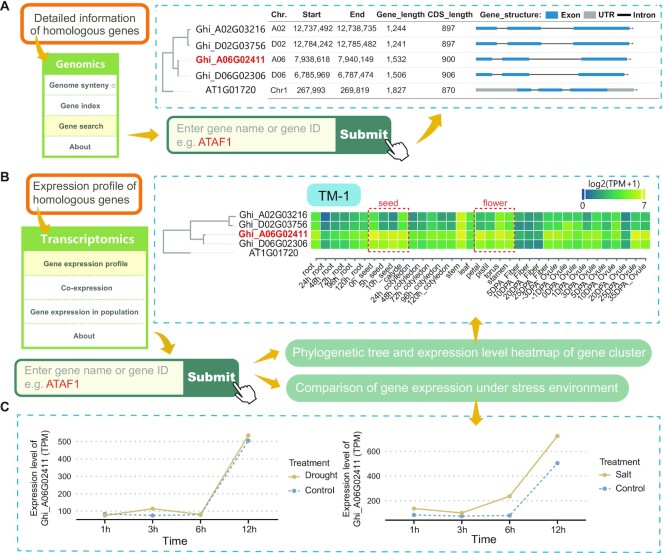
Usage of genomics and transcriptomics portals in CottonMD. (A and B) Gene structures (**A**) and expression patterns (**B**) of four *ATAF1* paralogs. The expression values are shown in Binary logarithm of TPM (transcript per million) + 1. Yellow and green represent high and low expression levels of genes in different tissues, respectively. (**C**) Expression levels of four *ATAF1* paralogs under drought and salt stresses. Yellow solid and blue dashed lines represent the gene expression levels under the control and stress, respectively.

***Case study 1*** *: Explore the gene structures and expression patterns of ATAF1 paralogs*. *ATAF1* encodes an *Arabidopsis thaliana* NAC transcription factor and plays important roles in plant adaptation to environmental stress and development (61). Currently, Ghi_A06G02411, an ortholog in *G. hirsutum*, has been validated to play a role in cotton adaptation to drought and salt stress (61). Using the Genomics and Transcriptomics portals of CottonMD, four *ATAF1* paralogs show similar gene structures and expression patterns (Figure 3A, B). Ghi_A06G02411 and Ghi_D06G02306 have higher expression than Ghi_A02G03216 and Ghi_D02G03756. Among them, Ghi_A06G02411 has the highest expression level, especially in flowers and seeds (Figure 3B), and Ghi_A06G02411 has higher expression under drought and salt stress than that of the control (Figure 3C). The above results suggest that Ghi_A06G02411 plays important roles in plant adaptation to drought and salt stress, which indicates that CottonMD can help researchers to understand the features and function of the interested genes.

At the population level, we collected whole genome sequencing (WGS) datasets of 4180 cotton accessions from previously published studies (Supplementary Table S5). After genotype imputation and filtering, a genetic variation panel including 12 903 345 SNPs and 1 381 741 InDels of 4180 cotton accessions was constructed (Supplementary Figure S1A). The information of genetic variations in this panel can be browsed in the Variation portal by the genomic region or gene ID. Based on this panel, the 4180 cotton accessions can be divided into eight groups, designated as G0–G7, which is similar to a previous study (Supplementary Figure S1B, C). To identify genomic regions during the domestication and selection process, four signals among subpopulations including genetic diversity (π), Tajima's *D* pairwise fixation statistic (*F*_ST_) and XP-CLR values were calculated. Sample information, population structure and selection signals can be acquired in the Population portal (Figure 2).

### Integration and association analysis of multi-omics data

Integration of genomic, transcriptomic and phenotypic information offers great opportunities of mapping candidate genes in loci associated with important traits and elucidating complex relationships across multiple genes and traits. In order to take full advantage of the genetic variations in CottonMD and reveal their effects on phenotypes and gene expression, we developed two modules, Single-locus and Multi-locus modules, in the Variation portal. The Single-locus module can provide detailed information of the variations including genomic distribution, variation type and allele frequency in a subpopulation based on the given genomic region or gene. More importantly, CottonMD associates the variations with traits and gene expression, which can greatly help understand the functional effects of alleles and genes. The Multi-locus module allows the joint analysis of two and more genes simultaneously to observe the effects of different loci on phenotypes, which can facilitate the understanding of interactions among different loci and dissection of the genetic basis for complex traits (***Case study 2***).

To identify the associations between variations and phenotypes and uncover their molecular mechanisms, we performed a joint analysis of multi-omics data with multiple statistical methods, including GWAS, eQTL mapping and TWAS. By an eQTL mapping of 44 616 expressed genes, 41 176 eQTLs were associated with 14 263 genes (eGenes), including 12 244 *cis*-eQTL and 28 932 *trans*-eQTL (Supplementary Table S6). GWAS of 20 phenotypes identified totally 27 loci with 1215 unique candidate variations significantly associated with 13 phenotypes, including 20 reported loci (55), suggesting a high repeatability of these loci and reliability of the method (Supplementary Table S7). Six fiber-related phenotypes were identified to be associated with the expression levels of 483 genes by TWAS (62) (Supplementary Table S8). In addition, SMR (Summary data–based Mendelian randomization analysis) and colocalization analysis were performed to detect the candidate genes associated with seven traits by integrating the GWAS and eQTL results. Totally, 23 candidate genes were associated with six phenotypes by SMR (Supplementary Table S9). *Cis*-eQTLs of 206 candidate genes were co-localized with the QTLs of 16 phenotypes by colocalization analysis (Supplementary Table S10). All variation-phenotype and gene-phenotype associations could be queried and visually browsed in CottonMD (***Case study 2***).

**Figure 4.**
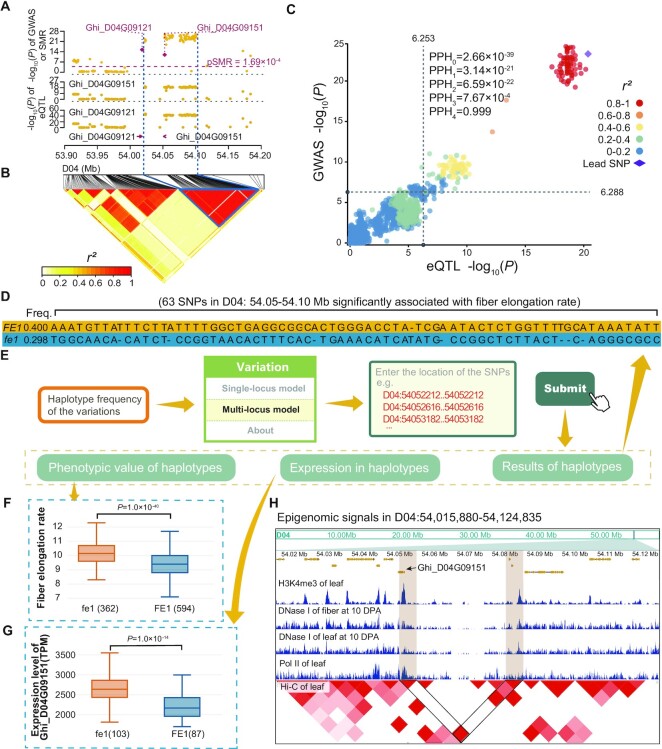
Case study of mining candidate genes based on multiple omics data: analyzing the *FE1* loci using CottonMD. (A and B) Manhattan plot (**A**) and LD heatmap (**B**) for GWAS, eQTL and SMR signals of *FE1* loci. The threshold value of SMR is 1.69 × 10^−4^ (grey dashed line). Yellow dots represent the GWAS significance of SNPs and red diamonds represent the SMR significance of genes. The purple diamond represents the SMR significance of the putative candidate gene (*PPR*) (red arrows) and red dots represent the GWAS significance of the putative causal variation (an InDel in the third exon of *PPR*). (**C**) Colocalization analysis of *FE1* loci. x and y axis represent the eQTL of Ghi_D04G09151 and GWAS significances of *FE* of variations, respectively. The purple diamond represents the lead variation and the dots with colors from blue to red represent the LD values related to the lead variation. (**D**) Haplotype frequency of the variations significantly associated with fiber elongation rate in the upstream of Ghi_D04G09151. Yellow and blue areas represent the frequency of the favorable (FE1) and unfavorable (fe1) haplotypes, respectively. (**E**) The pipeline of searching *FE1* locus in multi-locus variation modules. (E, F) Fiber elongation rate (**F**) and expression level (**G**) of Ghi_D04G09151 of accessions with FE1 (*n*= 594, blue boxes and violins) and fe1 (*n*= 362, orange boxes and violins) haplotypes. All significances are tested by the two-tailed Wilcoxon ranksum test. (**H**) JBrowser screenshot in *FE1* loci. The significance is tested by the two-tailed Wilcoxon rank sum test.

***Case study 2*** *: Analyze the effects on the cotton fiber elongation rate of FE1 loci*. We take the fiber elongation rate (FE) as an example to show how CottonMD facilitates systematic identification and analysis of the candidate genes. FE can represent the elongation ability of mature fiber cells (5), and three previously reported loci-*FE1*, *FE2* and *FE3* were significantly identified (Supplementary Figure S2; Supplementary Table S7). *FE1* on chromosome D04 explains the most phenotypic variance (5), and two candidate genes (Ghi_D04G09121 and Ghi_D04G09151) were identified by SMR (Figure 4A). Ghi_D04G09121 encodes pentatricopeptide repeat (PPR), which has been proved to be related to the development of cotton organs (63), and is mainly expressed in both the fiber and ovule using Transcriptomics portal (Supplementary Figure S3). Ghi_D04G09151 encodes tubulin alpha 2 (*GhTUA2*), which participates in several important cellular processes (64,65), and is mainly expressed in the fiber at 15 and 20 days post anthesis (DPA) (Supplementary Figure S4). Notably, two non-synonymous SNPs in Ghi_D04G09151 are not significantly associated with the trait (Supplementary Table S7); while 63 significant variations are enriched in the neighbor or 25–48 kb upstream region of Ghi_D04G09151 in a strong linkage disequilibrium (Figure 4A, B). Colocalization analysis indicates that eQTL of Ghi_D04G09151 and GWAS of FE shares the same causal variations (PPH_4_ = 0.99, Figure 4C). By using the Multi-locus module in the variation portal (Figure 4D, E), we grouped these variations into two haplotypes (the favorable *FE1* and unfavorable *fe1*) and the accessions with *fe1* haplotype showed the significantly higher FE and gene expression level (Figure 4F, G). Next, by combining the epigenetic data from CottonMD, we found that active histone signals (such as H3K4me1 and H3K4me3) were enriched in gene body as well as the 3-kb and 25–48 kb upstream regions of Ghi_D04G09151; the Pol II signal was enriched in the 3-kb upstream region; and the enrichment of DNase I signal was found in the 25–48 kb upstream region (Figure 4H), suggesting that these regions are likely the promoter and enhancer regions, correspondingly. The strong chromosomal interaction between the two regions indicates their regulatory relationship (Figure 4H). Therefore, we deduced that the variations in two regions affect the gene regulatory elements, leading to changes in gene expression and ultimately affecting the phenotype.

### Multi-omics analysis tools in CottonMD

We provided 10 common bioinformatic tools for 25 published cotton genomes in Tools portal of CottonMD, such as Blast (66) and GO/KEGG enrichment analysis (67,68), which can help quick analyses without switching between different databases or modules. In addition, we integrated the SNPmatch based on variations of 4180 accessions to facilitate the identification and management of germplasm resources (69).

## SUMMARY AND FUTURE DIRECTIONS

**Figure 5.**
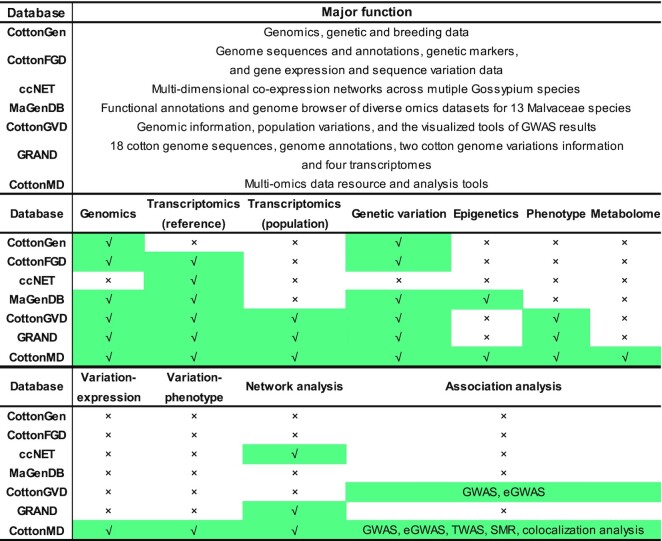
Summary of features distinguishing CottonMD from the published cotton database. Green regions indicate the omics data integrated in the corresponding database.

In this study, we mined and integrated the data of genomics, transcriptomics, genetic variation, phenotype, epigenome and metabonomics data in cotton. Subsequently, a multi-omics database for cotton biological study-CottonMD was constructed. Compared with other published cotton databases including CottonGen, CottonFGD, ccNET, MaGenDB, CottonGVD and GRAND, CottonMD has some attractive advantages as follows: (i) CottonMD is the first database to provide genome-wide variation-expression associations and variation-phenotype associations, which is important to mine the candidate variants or genes (Figure 5); (ii) CottonMD is the first database to provide online multi-omics analysis platform including SMR and colocalization analysis; (iii) CottonMD integrates and links the most comprehensive multi omics data at present and provides convenient searching tools (Figure 5), which can help researchers quickly acquire the related omics information; (iv) it provides multiple common bioinformatic analysis tools for 25 published cotton genomes, and all portals of the database support searching by gene name and gene ID of 25 published cotton and *Arabidopsis* genomes. There is no need to switch between different databases or modules.

In summary, CottonMD can provide an important resource and tools for the rapid identification of the candidate genes in the locus and to assist functional validation, as well as help to understand the mechanisms through which genetic variations affect gene expression and phenotype and to choose the optimal breeding strategy.

In the future, further development and advance of technologies will make more datasets available. Hence, integration of multi-omics data will be critical for genetic research. We will be integrating omics data from more accessions, more tissues and more omics and applying more powerful statistical methods to improve CottonMD.

## DATA AVAILABILITY

Sources of all datasets are described at supplemental materials and methods. And all datasets are made available at http://yanglab.hzau.edu.cn/CottonMD/download.

## Supplementary Material

gkac863_Supplemental_Files
