# Supplementary material for: CottonMD: a multi-omics database for cotton biological study
Source: Nucleic Acids Res. 2022 Oct 10;51(D1):D1446–56. doi: 10.1093/nar/gkac863 (PMC9825545; doi:10.1093/nar/gkac863)
Supplement: gkac863_Supplemental_Files [file gkac863_supplemental_files.zip › CottonMD_Supplementary_Figure_with_titles.pdf]

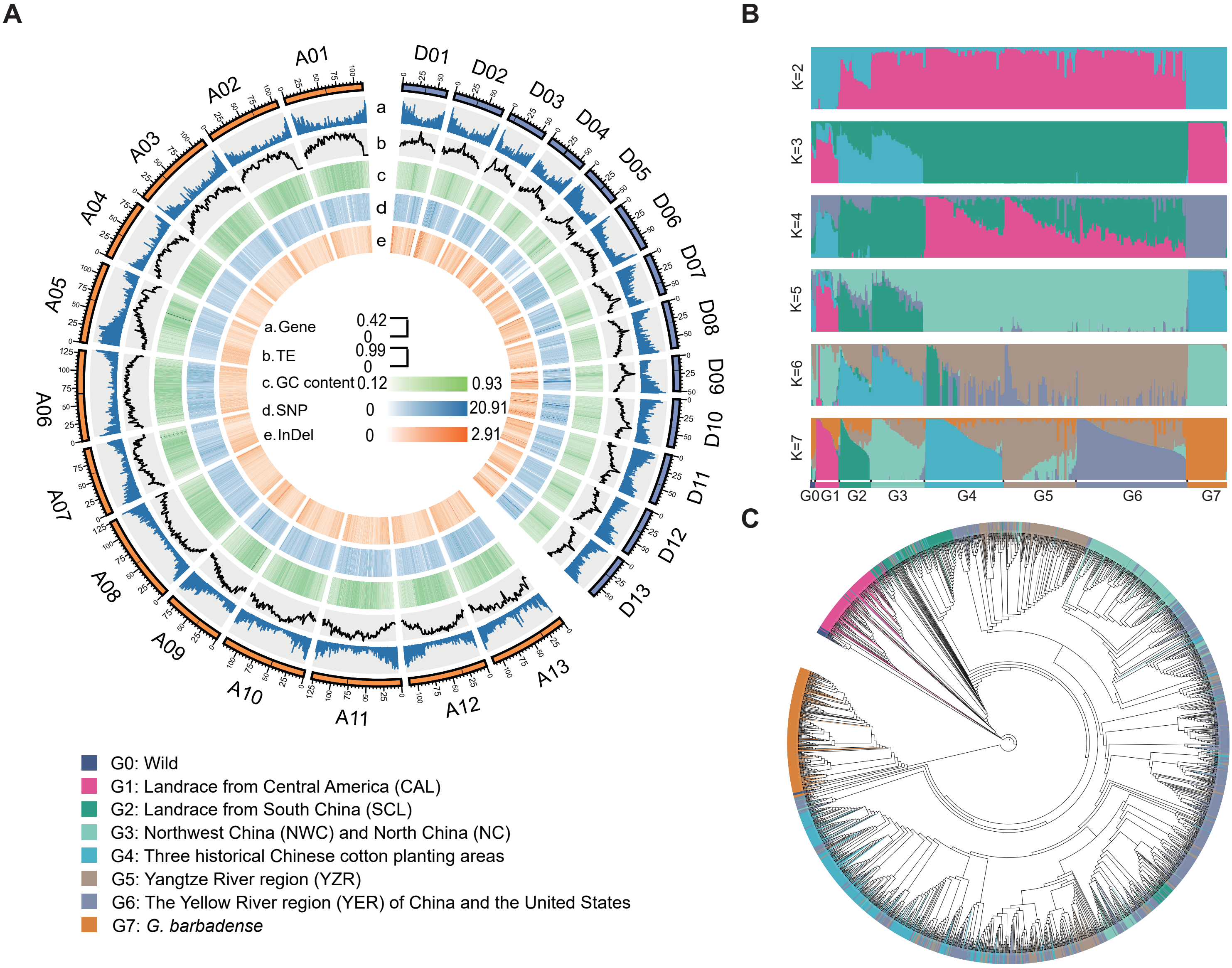

Figure S1: Construction and genetic diversity of 4,180 cotton accessions.

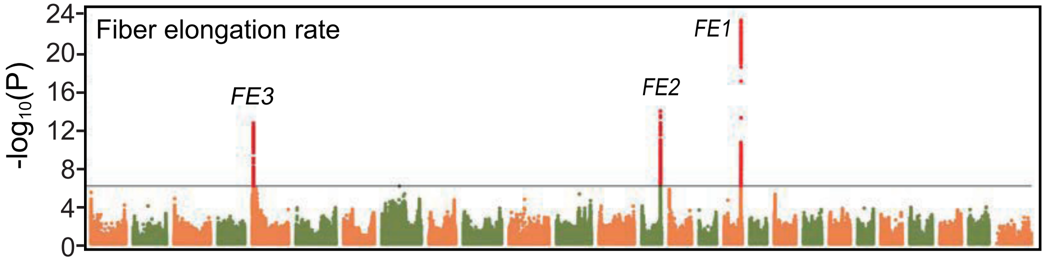

Figure S2: Manhattan plot of fiber elongation rate.

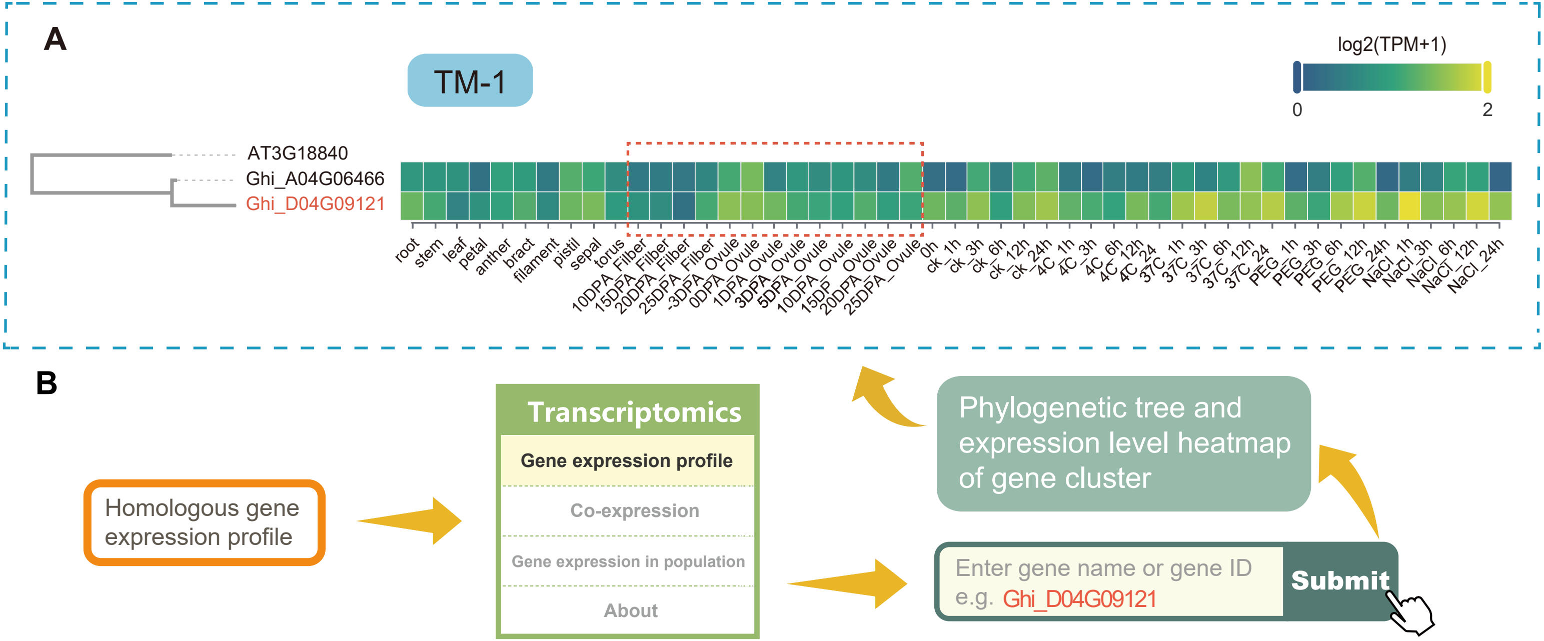

Figure S3: The transcriptomics analysis of Ghi_D04G09121 in CottonMD.

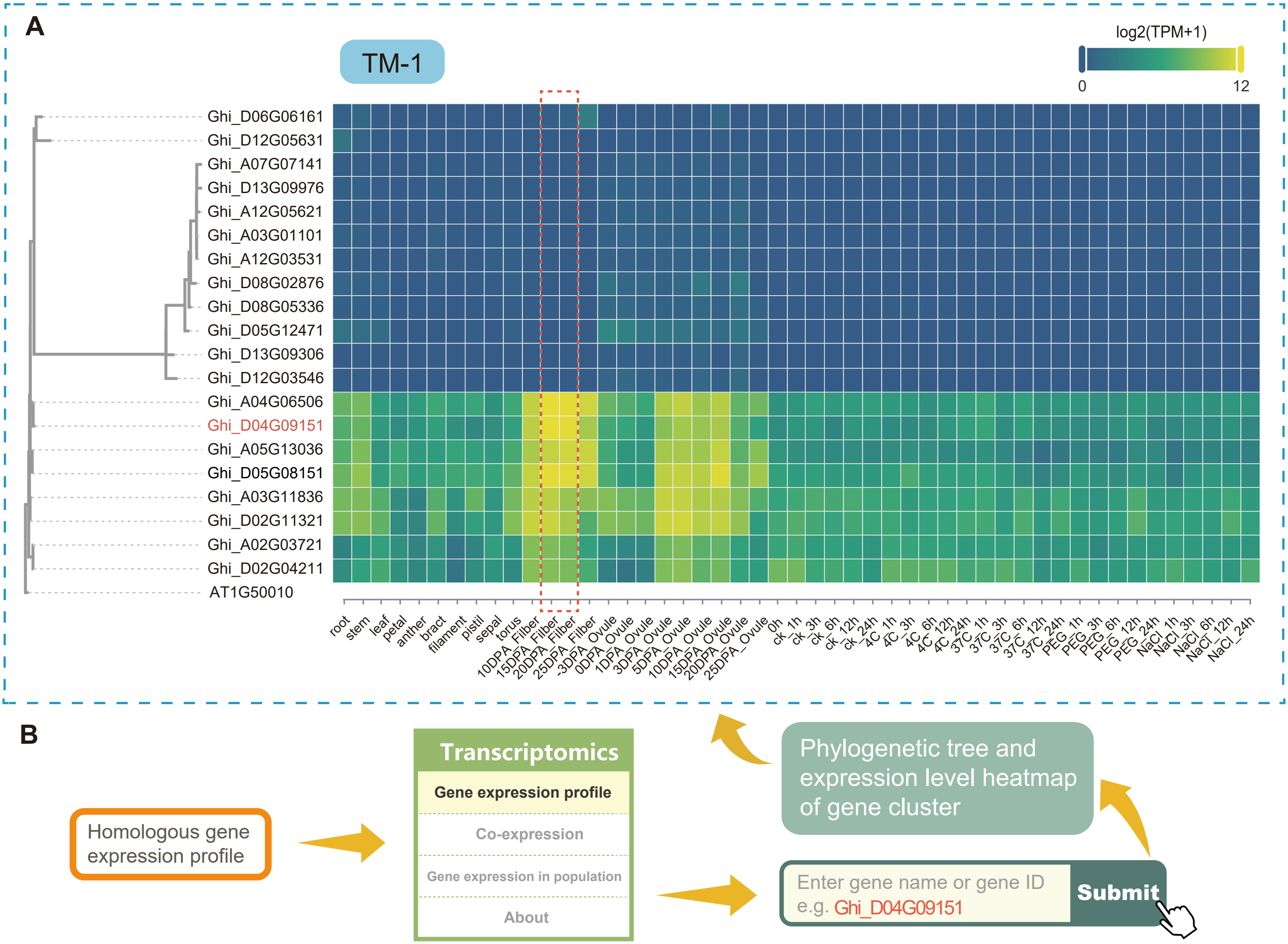

Figure S4: The transcriptomics analysis of Ghi_D04G09151 in CottonMD.
